# Chronic infection and metabolic stress: a converging axis driving hypertension and metabolic dysfunction-associated steatotic liver disease outcomes

**DOI:** 10.1093/ajh/hpag064

**Published:** 2026-06-22

**Authors:** Jannis Kountouras, Stergios A Polyzos, Elisabeth Vardaka, Christos Zavos, Michael Doulberis

**Affiliations:** Department of Medicine, Second Medical Clinic, Aristotle University of Thessaloniki, Ippokration Hospital, Thessaloniki, Macedonia, Greece; Department of Medicine, Second Medical Clinic, Aristotle University of Thessaloniki, Ippokration Hospital, Thessaloniki, Macedonia, Greece; First Department of Pharmacology, Medical School, Aristotle University of Thessaloniki, Thessaloniki, Macedonia, Greece; Department of Medicine, Second Medical Clinic, Aristotle University of Thessaloniki, Ippokration Hospital, Thessaloniki, Macedonia, Greece; Department of Nutritional Sciences and Dietetics, School of Health Sciences, International Hellenic University, Thessaloniki, Greece; Department of Medicine, Second Medical Clinic, Aristotle University of Thessaloniki, Ippokration Hospital, Thessaloniki, Macedonia, Greece; Department of Medicine, Second Medical Clinic, Aristotle University of Thessaloniki, Ippokration Hospital, Thessaloniki, Macedonia, Greece; Department of Gastroenterology & Hepatology, University Hospital Zurich, University of Zurich, Zurich, Switzerland; Division of Gastroenterology and Hepatology, Medical University Department, Kantonsspital Aarau, Aarau, Switzerland

**Keywords:** MASLD, chronic infection, arterial hypertension, metabolic stress, *Helicobacter pylori*, AMPK signaling, hypertension, blood pressure

## Abstract

Metabolic dysfunction-associated steatotic liver disease (MASLD) is increasingly recognized as a systemic cardiometabolic disorder arising from the convergence of metabolic stress, chronic low-grade (smoldering) inflammation, and vascular pathology. MASLD extends beyond hepatic fat accumulation, and is tightly linked to arterial hypertension and cardiovascular disease, with arterial hypertension acting as a disease modifier that accelerates fibrosis progression and deteriorates long-term outcomes. Emerging evidence suggests that chronic infections, particularly *Helicobacter pylori* infection, may further amplify this cardiometabolic continuum by sustaining inflammatory signaling, oxidative stress, insulin resistance, and endothelial damage. These mechanisms substantially overlap with crucial pathogenic pathways implicated in MASLD progression and hypertension onset. At the molecular level, AMP-activated protein kinase (AMPK) signaling represents a key integrative axis linking energy homeostasis, inflammation, and blood pressure regulation. Pharmacological AMPK activation, exemplified by imeglimin targeting presenilin enhancer-2, highlights the translational potential of therapies addressing hepatic and vascular injury simultaneously. This perspective summarizes clinical and mechanistic evidence supporting an integrated, systems-based framework in which chronic infection and metabolic stress converge along a shared axis to drive hypertension and MASLD. It underscores the need to incorporate metabolic regulation, cardiovascular risk modification, and selected infectious determinants into future risk stratification models and therapeutic strategies.

## Graphical abstract

**Graphical Abstract:**
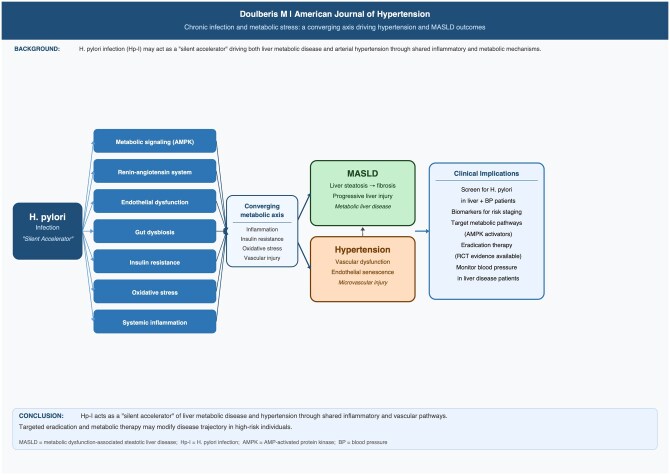

## Introduction

Chronic low-grade (smoldering) inflammation and metabolic stress are increasingly recognized as central, shared drivers of cardiometabolic diseases, including arterial hypertension, atherosclerosis, and metabolic liver disorders. Perpetual inflammatory microenvironment—arising from metabolic overload, vascular dysfunction, or chronic infection—reprograms immune and metabolic signaling, with downstream effects on vascular tone, insulin responsiveness, and long-term end-organ susceptibility.^1^

This systemic inflammatory milieu provides a biological substrate through which cardiovascular and metabolic diseases converge, rather than evolve as distinct clinical entities. Such integration is particularly evident in disorders characterized by sustained metabolic stress and sustained immune activation, where subtle but chronic perturbations amplify disease progression over time.^2^

Metabolic dysfunction-associated steatotic liver disease (MASLD) has emerged as the most prevalent chronic liver disorder worldwide, impacting over 30% of the world’s population.^3^ It represents the hepatic manifestation of a systemic cardiometabolic continuum, most commonly expressed as metabolic syndrome (MetS). MetS, together with overweight and obesity and its advanced hepatic form—metabolic-associated steatohepatitis (MASH)—now represents the leading cause of liver cirrhosis, liver transplantation, hepatocellular carcinoma, and liver-related mortality.^4^

MASLD encompasses a spectrum of liver injury driven by metabolic stress and is closely associated with insulin resistance (IR) and genetic susceptibility, although its pathological changes are unrelated to alcohol.^5^

Beyond hepatic steatosis, MASLD is strongly linked to arterial hypertension, cardiovascular disease (CVD), and adverse long-term outcomes, including fibrosis progression and hepatic decompensation.^6–9^ Epidemiological and longitudinal studies indicate that hypertension not only coexists with MASLD but actively accelerates histological disease progression and worsens prognosis, underscoring its relevance to hepatologists, cardiologists, and primary care providers alike. In this context, MASLD represents not merely a liver-specific disorder but a nodal point where metabolic stress, vascular dysfunction, and inflammatory signaling intersect.^10^^,^^11^

Apart from metabolic and hemodynamic factors, chronic infections have been increasingly acknowledged as long-term modulators of systemic inflammation and cardiometabolic risk.^1^^,^^12^ Among chronic infections, *Helicobacter pylori* infection (*Hp*-I) has been increasingly linked to features of the MetS, arterial hypertension, and MASLD.

Beyond summarizing current evidence, this perspective article introduces a novel conceptual framework where chronic infection, for instance *Hp*-I, serves as a “silent accelerator” that synchronizes hepatic and vascular injury by perturbing common metabolic nodes, including the AMP-activated protein kinase (AMPK) and renin-angiotensin-aldosterone system (RAAS) pathways.

## Metabolic stress and signaling convergence in MASLD

At the molecular level, metabolic signaling pathways that integrate energy balance, inflammation, and vascular homeostasis are central to this convergence. AMPK functions as a key metabolic hub, exerting protective effects against IR, oxidative stress, and hypertension.

Impaired AMPK signaling has been implicated in both vascular dysfunction and MASLD pathogenesis. Activation of AMPK improves insulin sensitivity, suppresses hepatic lipid synthesis, and enhances fatty acid oxidation, thereby helping to slow the progression of MASLD.^13^ Mechanistically, AMPK reduces MASLD by suppressing hepatic de novo lipogenesis, increasing fatty acid oxidation, and preserving mitochondrial function and integrity in adipose tissue.^14^ These effects support the feasibility of AMPK-targeted therapeutic strategies for MASLD.^15^ Collectively, AMPK plays a central role in regulating cellular energy metabolism and mitigating oxidative stress, and has emerged as a key regulatory protein capable of counteracting cytotoxicity, attenuating inflammation, and inhibiting fibrotic processes. As such, AMPK represents a promising therapeutic target for addressing the major pathogenic mechanisms underlying MASLD.

Beyond its substantiated role in energy homeostasis and lipid metabolism, AMPK serves as an upstream activator of nuclear factor erythroid 2-related factor 2 (NRF2), a master transcription factor orchestrating the cellular antioxidant response and endogenous repair capacity. From a mechanistical perspective, AMPK promotes NRF2 nuclear translocation by direct phosphorylation at Ser448 and by inhibiting the GSK-3β/βTrCP degradation axis, thereby inducing the expression of cytoprotective enzymes—including heme oxygenase-1 (HO-1) and NAD(P)H quinone oxidoreductase 1 (NQO1)—which constrain oxidative damage and attenuate hepatocellular integrity.^16^^,^^17^ In the setting of MASLD, NRF2 activation has been reported to improve hepatic steatosis, reduce stellate cell activation, and mitigate fibrogenesis, highlighting its key role to hepatic cytoprotection.^18^ Nevertheless, in the context of chronic low-grade (smoldering) inflammation—as observed in both MASLD and *Hp*-I-perpetual NF-κB activation suppresses NRF2 signaling through competitive chromatin remodeling at antioxidant response element (ARE) loci, resulting in a functionally compromised antioxidant defense system.^19^ Notably, *Hp*-I has been displayed to directly downregulate NRF2 expression and hamper its nuclear translocation, thereby amplifying oxidative injury and promoting epithelial dysfunction—a mechanism that may extend beyond the gastric mucosa to contribute to systemic metabolic perturbations.^20^ Furthermore, NRF2 activity has been identified as a key player of hepatic progenitor cell (HPC) fate: NRF2 downregulation promotes HPC activation and differentiation, a response that—although initially regenerative—renders maladaptive under sustained inflammatory conditions, promoting ductular reaction and progressive fibrosis.^21^ Consistent with this, HPC activation and ductular reaction have been described as independent histological indicators of disease activity and fibrosis severity in patients with MASLD.^22^ Taken together, these observations position the AMPK-NRF2 axis as a central node linking chronic inflammation, impaired endogenous repair, and fibrosis progression in MASLD, with *Hp*-I potentially amplifying this pathway through convergent suppression of NRF2-mediated cytoprotection.

Recent experimental evidence highlighting pharmacological AMPK activation—such as via the novel antidiabetic agent imeglimin, which targets presenilin enhancer 2 (PEN2)—underscores the therapeutic relevance of this pathway in alleviating hepatic lipotoxicity and metabolic injury.^23^ Viewed through a broader lens, AMPK represents a mechanistic intersection where chronic infection-driven inflammation, metabolic stress, and hypertension may jointly shape MASLD progression.^24^^,^^25^ Importantly, AMPK suppression in vascular endothelial cells concurrently reduces endothelial nitric oxide synthase (eNOS)-dependent nitric oxide bioavailability, directly linking *Hp*-driven inflammation to endothelial dysfunction and hypertension as a mechanistically distinct yet convergent output of the same mechanism.^26^^,^^27^

## Chronic infection as a modifier of metabolic and vascular injury

Beyond metabolic and hemodynamic stressors, chronic low-grade infections have emerged as sustained modifiers of systemic inflammation and cardiometabolic risk.^1^  *Hp*-I is highly prevalent worldwide, affecting over 4.4 billion people.^28^ Although direct causal relationship between *Hp*-I and MASLD has not been proven, yet, increasing evidence suggests a close association between *Hp*-I and MASLD, the mentioned hepatic manifestation of MetS.

Mice inoculated with *Hp* and fed with high-fat diet, developed more steatohepatitis, glucose, lipids, liver enzyme profile and waist circumference than the *Hp*-negative counterparts.^29^ Likewise, *Hp*-I could promote liver fibrosis in a murine model of steatohepatitis.^30^ Regarding human patients, *Hp* has been repeatedly identified in liver parenchyma of patients with MASLD and IgG seropositivity has been associated with MASLD diagnosis.^29^ Bariatric patients with *Hp*-I were characterized by statistically more advanced liver fibrosis, steatosis, lobular inflammation and MetS components including arterial hypertension compared to negative ones.^31^ Moreover, multiple meta-analyses support further this association,^29^^,^^32^ including a meta-analysis demonstrating a direct correlation between Hp-I and the presence of moderate-to-severe MASLD.^33^

Experimentally, it has been proposed that *Hp*-related inflammatory signaling may reduce hepatocyte glycogen levels via the c-Jun N-terminal kinase pathway, suppressing key glycolytic genes and accelerating lipolysis; however, direct human mechanistic evidence for this specific pathway in MASLD remains limited.^34^ Notably, MASLD is associated with an approximately 1.6-fold increased risk of hypertension, and multiple cross-sectional studies have demonstrated that both the presence and severity of MASLD are significantly correlated with arterial prehypertension and arterial hypertension.^35^^,^^36^

Moreover, cytokeratin-18 (CK-18) is a well-established marker of hepatocyte-specific apoptosis and serves as a valuable noninvasive biomarker for assessing the severity of hepatic inflammation, steatosis, and fibrosis.^37^ Elevated CK-18 expression has been observed during atherosclerotic plaque formation and in patients with hypertension and MASLD.^38^ Serum CK-18 is widely used to detect MASH and to evaluate fibrosis severity^39^; its fragments, M30 (CK18-M30) and M65 (CK18-M65), are considered indicators of hepatocellular injury in MASH.^40^ Patients with MASLD exhibited markedly higher CK18 levels than those with simple steatosis, and CK18 correlated positively with ALT, supporting its utility as a biochemical indicator of hepatocellular injury. Circulating CK-18 fragments have attracted increasing attention as a potential tool for distinguishing MASH from simple steatosis.^41^ Mechanistically, CK18 release reflects both hepatocyte apoptosis and necrosis: CK18 M30 represents caspase-mediated apoptosis, whereas CK18 M65 reflects total cell death (apoptotic + necrotic). This distinction is particularly valuable for differentiating MASH from simple steatosis and for assessing disease severity.^42^

Furthermore, CK-18 immunoreactivity is significantly increased in the foveolar epithelium of *Hp*-positive gastritis compared with *Hp*-negative gastritis and healthy controls, with particularly high expression observed in patients infected with cytotoxin-associated gene A (CagA)-positive *Hp* strains.^43^ Corroborating this, sero-epidemiological data link the presence of CagA and VacA (vacuolating cytotoxin A) virulence factors specifically to MASLD pathogenesis, reinforcing the hypothesis that eradication of virulent *Hp* strains may confer hepatic benefit beyond simple reduction of bacterial load.^44^ These findings suggest that *Hp*-I-driven apoptotic signaling, particularly via CagA-positive strains, may directly contribute to hepatocyte CK-18 release in the context of MASLD, providing a plausible mechanistic link between *Hp*-related inflammation and increased circulating CK-18 fragments as markers of disease activity. Direct clinical studies measuring CK-18 fragments specifically in patients with *Hp*-positive MASLD are, however, currently lacking and are warranted to formally validate this relationship.

Additionally, MetS-related features, including dyslipidemia and type 2 diabetes mellitus, in the presence of *Hp*-I are associated with a greater propensity for MASLD development and progression.^7^

Notably, in patients with type 2 diabetes, *Hp*-I acts as a distinct risk enhancer for MASLD development, suggesting that combined glycemic management and *Hp* eradication may reduce MASLD prevalence in this subpopulation.^45^ Emerging clinical evidence supports *Hp* eradication as a possible modifier of MASLD-related metabolic parameters. In this respect, a relevant meta-analysis of 4 RCTs encompassing 453 patients demonstrated that *Hp* eradication significantly reduced hepatic steatosis and circulating TNF-α levels in patients with NAFLD (currently defined as MASLD) compared to lifestyle modification alone.^32^ Additionally, a randomized controlled trial by Yu et al^46^ further exhibited that successful *Hp* eradication in 200 patients with NAFLD led to significant amelioration in fasting blood glucose, HOMA-IR, triglycerides, BMI, hs-CRP (high-sensitivity C-reactive protein), and IL-6 at 1 year of follow-up. These findings provide preliminary but clinically relevant evidence that *Hp* eradication may attenuate both the inflammatory and metabolic drivers of MASLD progression.

Furthermore, emerging evidence indicates that persistent active *Hp*-I is independently associated with MASLD development and can accelerate liver damage and fibrosis progression.^47^ However, important limitations must be acknowledged: the available trials are few in number, involve short follow-up periods, rely predominantly on surrogate metabolic endpoints, and lack histological fibrosis data as primary outcomes. Whether *Hp* eradication can meaningfully alter the natural history of MASLD—particularly by reducing fibrosis progression—remains to be established in adequately powered prospective studies with standardized endpoints.

Accumulating clinical and experimental evidence indicates that *Hp*-associated inflammatory and metabolic perturbations may synergistically contribute not only to the development of MASLD but also to its progression via intersecting cardio-cerebrovascular and hepatic pathways.^6^^,^^7^ Active *Hp*-I has been linked to MetS-related systemic conditions, including CVD and neurodegenerative disorders, which represent the endpoint of MetS.^48^ In this context, hypertension—an important risk factor linking *Hp*-I and MetS-related CVD—emerges as a principal contributor to mortality associated with *Hp*-I and MetS-related ischemic heart disease, stroke, and neurodegenerative disorders.^49^  *Hp*-I and MetS-associated arterial hypertension may contribute to MetS-related brain neurodegenerative disorders, through blood-brain barrier disruption, exacerbation of amyloid disorders, and reduced function of the cerebral blood vessels, including decreased cerebral blood flow, altered brain autoregulation, and compromised neurovascular coupling.^49^

Previous work of our study group^7^ has emphasized the role of *Hp*-related MetS as a systemic driver of arterial hypertension, proposing therefore that chronic infection may serve as a disease modifier rather than a pure “co-incidence.” Based on this rational, the present perspective expands the concept toward MASLD as a central cardiometabolic hub.

## Mechanistic pathways linking Hp, MASLD, and arterial hypertension

From a clinical and translational perspective, these converging pathways underscore MASLD as a systemic disease entity in which metabolic stress, chronic infection, and vascular dysfunction jointly shape disease trajectory and outcomes.

In this context, both imeglimin and AMPK axis activation may exert antihypertensive effects that contribute to the prevention and attenuation of MASLD development and progression. AMPK activation is known to confer protective effects against,^50^ whereas impaired AMPK signaling has been implicated in the pathogenesis of hypertension and its associated complications.^26^

Recent evidence indicates that arterial hypertension, increases disease progression, worsens outcomes, and promotes histological fibrosis progression in MASLD, underscoring its relevance for hepatologists, cardiologists, and primary care providers.^51^ Moreover, emerging data suggest that a considerable proportion of patients with MASLD and advanced (F3) fibrosis progress to (F4) cirrhosis with later decompensation, accompanied by significant adverse outcomes.^52^

The principal molecular and pathophysiological mechanisms linking *Hp*-associated metabolic stress, MASLD, arterial hypertension are summarized in Table 1. The proposed mechanisms linking *Hp* and MetS-related MASLD may promote arterial hypertension by inducing:

**Table 1:** Integrated mechanistic pathways linking Hp infection, MASLD, and arterial hypertension.

Pathway	Hp-related trigger	Effect on MASLD	Contribution to AH	Evidence base
Systemic inflammation	TNF-α, IL-6, chronic immune activation, neutrophil activation	Hepatic inflammation, fibrosis progression	RAAS activation, vascular inflammation	**E, C**
Oxidative stress	ROS generation, mitochondrial dysfunction	Lipotoxicity, hepatocyte injury	Endothelial dysfunction	**E, C**
Insulin resistance	Inflammatory signaling, metabolic dysregulation	Steatosis, MASH progression	Sympathetic activation, sodium retention	**E, C**
Gut dysbiosis	Altered microbiota, increased intestinal permeability	Endotoxemia, liver inflammation	Low-grade inflammation, vascular stiffness	**E, C**
Endothelial dysfunction	ADMA elevation, reduced NO bioavailability	Impaired hepatic microcirculation	Increased vascular resistance	**C, I**
RAAS activation	Cytokine-mediated angiotensinogen upregulation	Fibrosis progression	Sustained hypertension	**C, I**
AMPK impairment	Metabolic stress, chronic inflammation	Reduced lipid oxidation, mitochondrial dysfunction	Loss of vasoprotective effects	**E, I**

**Evidence base:** (*E) supported by experimental* in vitro *or animal model data; (C) supported by clinical or epidemiological studies; (I) inferred from indirect mechanistic associations. Where multiple designations apply, all are listed*.

Abbreviations: ADMA, asymmetric dimethylarginine; AH, arterial hypertension; AMPK, AMP-activated protein kinase; Hp, *Helicobacter pylori*; IL-6, interleukin-6; MASLD, metabolic dysfunction-associated steatotic liver disease; MASH, metabolic dysfunction-associated steatohepatitis; NO, nitric oxide; RAAS, renin-angiotensin-aldosterone system; ROS, reactive oxygen species; TNF-α, tumor necrosis factor-alpha.

### Systemic inflammation

MASLD has been closely associated with systemic inflammatory responses, characterized by elevated circulating levels of pro-inflammatory cytokines, such as IL-6 and TNF-α.^4^  *Hp*-related increases in TNF-α and IL-6, contribute to the development of hypertension. These *Hp-*related cytokines have been shown to regulate components of the RAAS, particularly by enhancing angiotensinogen production in the liver, thereby promoting systemic and local angiotensin II formation and angiotensin II-dependent hypertension.^37^ In addition, MASLD-induced systemic inflammation may facilitate inflammatory cell infiltration within the vasculature, kidneys, and adipose tissue, further accelerating the onset and progression of hypertension.^1^^,^^11^ However, further studies are needed to elucidate these mechanisms.

Beyond *Hp*-related cytokine release, visceral adipose tissue is immnunologically active by secreting spatially relevant source of pro-inflammatory mediators—including TNF-α, IL-6, and free fatty acids—that are delivered directly to the liver via the portal circulation, thereby amplifying hepatic inflammatory signaling and contributing to hepatocellular injury and steatohepatitis progression in MASLD.^53^ Parallelly, dysfunctional perivascular adipose tissue (PVAT)—a metabolically active depot in close anatomical proximity to blood vessels—loses its physiological anti-inflammatory and vasoprotective properties in obesity and MetS, instead releasing leptin, resistin, and pro-inflammatory cytokines that promote local vascular inflammation, endothelial dysfunction, and heightened vascular tone.^54^ These adipose-derived inflammatory inputs act synergistically with *Hp*-I-driven immune activation, compounding cytokine burden at the hepatic and vascular level and further sustaining the cardiometabolic continuum underlying MASLD and arterial hypertension.

### Enhanced oxidative stress

MASLD has been strongly associated with oxidative stress.^55^ In this context, reactive oxygen species may contribute to *Hp*-related extragastric manifestations of MetS, including atherosclerosis implicated in hypertension.^12^ Accordingly, enhanced oxidative stress may represent a key mechanistic link among *Hp*-I, MASLD, and hypertension.

### Insulin resistance

Growing evidence supports a potentially causal relationship between MASLD and *Hp*-related IR. Many studies have confirmed the association between chronic *Hp*-I and IR, suggesting that this infection may represent an independent risk factor for the development of IR in patients with MASLD.^56^  *Hp*-I contributes to IR, a hallmark of MetS and a key driver in the etiopathogenesis of atherosclerosis and hypertension-related target-organ damage.

Mechanistically, IR promotes the release of excess free fatty acids from adipose tissue, leading to ectopic fat accumulation—including perivascular and renal sinus fat—which in turn increases the risk of hypertension.^57^

### Gastrointestinal dysbiosis

*Hp*-associated gastrointestinal dysbiosis has been linked to MetS-related systemic disorders, including hypertension and MASLD.^58^ Research involving animal models that alter gut microbiota, along with observational studies in patients with MASLD, indicates that dysbiosis of the gut microbiota plays a role in the development of MASLD.^59^ MASLD connects with gastrointestinal dysbiosis, suggesting that dysbiosis may contribute to disease development and progression. Dysbiosis raises intestinal permeability to bacterial toxins and elevates liver exposure to harmful compounds, leading to heightened liver inflammation and fibrosis. The existence of *Hp* is linked to heightened intestinal permeability.^60^ It has been proposed that the processes underlying MASLD development related to *Hp*-associated dysbiosis may involve *Hp*-mediated mucosal permeability, progression of intestinal dysbiosis, and translocation of bacterial endotoxin (lipopolysaccharides) via the portal vein to the liver—though this mechanistic chain remains to be formally established in human studies. Based on indirect associations, *Hp*-related MetS microbiota dysbiosis has been suggested to contribute to MASLD pathogenesis and its systemic complications, though direct causal evidence in humans is currently lacking. Preliminary animal data suggest that fecal microbiota transplantation may attenuate MASLD features, though clinical translation of these findings remains, again, to be established.^31^

Specifically, fecal microbiota sequencing studies have demonstrated reduced microbial richness and diversity in patients with MASLD, and increased *Bacteroides* and decreased *Prevotella*, taxa closely associated with short-chain fatty acid production.^61^ These microbiota alterations have been proposed to further promote arterial hypertension through systemic inflammatory signaling, though direct mechanistic evidence in humans is limited. Alongside gastrointestinal dysbiosis, analyses of fecal microbiome profiles in patients with MASLD have shown enrichment of genes encoding proteins involved in lipopolysaccharide biosynthesis, which can compromise intestinal barrier integrity.^61^ This disruption may facilitate the translocation of microbial-associated molecular patterns and gut-derived metabolites, thereby triggering intestinal and hepatic inflammatory responses. The potential contribution of *Hp* translocation to this process has been proposed based on experimental data, though its clinical relevance in patients with MASLD remains to be confirmed.^62^ Furthermore, circulating lipopolysaccharide levels are significantly elevated in patients with MASH,^63^ potentially promoting systemic inflammation and contributing to the development of hypertension.

Moreover, emerging experimental evidence suggests that gut dysbiosis may influence neutrophil activation and NETs formation, although the specific contribution of *Hp*-associated dysbiosis to this process in MASLD has not yet been directly demonstrated.^64^ Aberrant NETs activity has been reported in the context of *Hp*-I^65^ and associated with MetS-related disorders such as MASLD, arterial hypertension, and CVD, although a direct mechanistic link between *Hp*-driven NETs and MASLD progression remains inferential.^66^ NETs triggered by hepatic fat accumulation can promote MASLD progression through augmentation of pro-inflammatory responses, thereby suggesting the involvement of neutrophils in MASLD and MASH. Understanding the complex roles of neutrophils within the liver’s unique microenvironment offers insights into novel therapeutic strategies and highlights the need for further research into neutrophil-targeted interventions for managing MASLD and MASH.^67^

Likewise, the neutrophil-albumin ratio (NAR) combines data on chronic inflammation and metabolic conditions and plays a crucial role in clinical settings. The NAR is a noninvasive predictor of MASLD.^68^ It demonstrates the best predictive performance for the risk of MASLD.^69^ In addition, the NAR demonstrates a more significant predictive value for all-cause and CVD mortality in patients with MASLD.^69^ Notably, emerging evidence indicates that NAR is associated with hypertension.^69^

The connection between the NAR-associated with *Hp*-I and MetS and heightened inflammation supports the progression of hepatic IR and lipid storage,^70^^,^^71^ and an elevated NAR might also promote neutrophil-associated processes, such as the formation of NETs, causing injury to liver cells and fibrosis, which could promote the progression of MASLD to MASH.^70^^,^^72^ Direct epidemiological evidence for this relationship was recently provided by He et al^73^ who demonstrated in a large cross-sectional mediation analysis of 26,245 participants that *Hp*-I was independently associated with MASLD, and that NAR statistically mediated a significant proportion of this association-supporting NAR as a biologically relevant intermediate linking *Hp*-related systemic inflammation to hepatic metabolic injury. These findings support systematic screening for *Hp*-I in MASLD populations to better evaluate individual risk, allowing for early intervention and prevention.

### Increased vasoconstriction and reduced vasodilation

Asymmetrical dimethylarginine (ADMA), a marker of endothelial dysfunction, is associated with vascular flow abnormalities in hypertension. Circulating ADMA concentrations correlate with the prevalence of MetS,^74^ and patients with MASLD exhibit elevated ADMA levels even in the absence of traditional cardiovascular risk factors.^75^ In addition, MASLD is associated with impaired endothelial nitric oxide synthase activity in circulating platelets,^27^ which may reduce nitric oxide bioavailability and compromise nitric oxide-dependent vasodilation.

Systemic inflammation in MASLD may further activate the RAAS, resulting in increased production of angiotensin II and other RAAS components, thereby promoting vasoconstriction. Dysregulation and overactivation of the RAAS play a central role in the development of hypertension and are linked to increased morbidity and mortality in MetS-related obesity and CVD, making the RAAS a key therapeutic target in the management of hypertension.^7^ Moreover, MASLD contributes to arterial stiffness, a process that impairs vascular compliance and promotes the development of hypertension. Similarly, *Hp*-I is associated with increased arterial stiffness—an early marker of atherosclerosis linked to hypertension and an independent predictor of CVD and all-cause mortality.^7^ Collectively, these findings suggest that MASLD may directly enhance vasoconstrictive mechanisms and promote arterial hypertension, although further mechanistic studies are required.

Beyond classical hemodynamic mechanisms, cellular senescence and microvascular inflammation represent additional pathogenic layers through which *Hp*-I may amplify vascular dysfunction in MASLD. *Hp*-I promotes inflammation-associated senescence in both gastric and extragastric cells, generating a senescence-associated secretory phenotype (SASP) that sustains chronic local and systemic inflammation and contributes to vascular injury beyond the gastric compartment.^76^^,^^77^ In MASLD, senescent hepatocytes display paracrine profibrogenic effects on hepatic stellate cells through SASP-derived mediators—including PDGF (platelet-derived growth factor) and TGF-β (transforming growth factor-β)—thereby accelerating fibrogenesis in a manner tightly correlated to fibrosis severity.^78^^,^^79^ At the hepatic microvascular environment, sinusoidal endothelial cell dysfunction consists an early event that precedes overt inflammation and fibrosis, disrupting intrahepatic microcirculation and reducing nitric oxide bioavailability.^80^ Parallelly, *Hp*-I is independently associated with systemic endothelial dysfunction, evidenced by impaired flow-mediated vasodilation and elevated vascular inflammatory markers, suggesting that *Hp*-related microvascular injury extends beyond the gastric vasculature to amplify the cardiometabolic burden in susceptible individuals.^81^

### Genetic and epigenetic modifications

Among the most extensively studied variants, the *PNPLA3 I148M* polymorphism (*rs738409 C > G*) shows a strong association with hepatic fat accumulation and increased disease severity.^82^^,^^83^ This variant impairs *PNPLA3* enzymatic activity, promoting macrovesicular steatosis and increasing the risk of hepatocellular carcinoma, particularly in individuals with MetS-related risk factors such as obesity.^83^ Notably, the combination of active *Hp*-I and the PNPLA3 rs738409 variant has been shown to synergistically associate with more severe MASLD phenotypes, suggesting a gene-infection interaction that may amplify disease risk beyond either factor alone.^84^ Additional genetic contributors include the *TM6SF2 E167K* variant, which reduces very-low-density lipoprotein secretion and enhances hepatic lipid retention, as well as variants in *TERT*, which have been linked to fibrosis progression.^83^

Gene-metabolite-disease interaction network analyses demonstrate shared molecular signatures linking MASLD and hypertension. Despite evidence supporting their clinical association, data on the genetic basis connecting MASLD and hypertension remain limited. Bioinformatic studies have identified the *AGTR1* (angiotensin II receptor type 1) gene—also implicated in *Hp*-I and MetS—as a potential contributor to signaling pathways involved in MASLD pathogenesis. The gain-of-function A1166C (rs5186) variant of *AGTR1* has been identified as a strong predictor of incident MASLD and hypertension.^85^

Although previous clinical trials failed to demonstrate a clear benefit of angiotensin receptor blockers (ARBs) in reducing fibrosis among patients with MASLD,^86^ individuals carrying the *AGTR1* A1166C variant may represent a MASLD subtype that could derive greater benefit from personalized ARB therapy.

**Figure 1:**
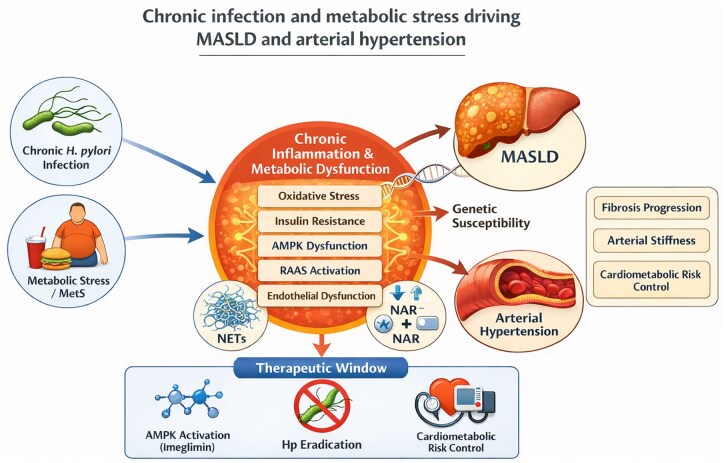
Converging pathways linking chronic infection, metabolic stress, hypertension, and MASLD progression. Chronic metabolic stress and persistent low-grade infection, particularly *Helicobacter pylori*, promote a sustained pro-inflammatory milieu characterized by systemic inflammation, oxidative stress, insulin resistance, endothelial dysfunction, and immune activation. These processes converge on shared molecular and neurohumoral pathways—most prominently AMP-activated protein kinase (AMPK) signaling and the renin-angiotensin-aldosterone system—thereby fostering arterial hypertension and accelerating hepatic injury and fibrosis progression in metabolic dysfunction-associated steatotic liver disease (MASLD). Innate immune effectors, including neutrophil extracellular traps (NETs), along with inflammation-based composite biomarkers such as the neutrophil-to-albumin ratio (NAR), reflect the interface between immune dysregulation, metabolic stress, and vascular injury. Genetic susceptibility factors further modulate this cardiometabolic-hepatic axis, shaping individual risk profiles and disease trajectories. Collectively, AMPK emerges as a central integrative node linking energy homeostasis, inflammation, and vascular regulation, highlighting potential targets for integrated cardiometabolic and hepatic intervention.

Collectively, these interrelated mechanisms illustrate how metabolic stress, chronic *Hp*-I-related inflammation, and vascular dysfunction converge to promote MASLD progression and arterial hypertension, forming a self-perpetuating cardiometabolic axis (Figure 1). Among these, systemic inflammation and insulin resistance constitute the primary hubs, while oxidative stress, gut dysbiosis, RAAS activation, endothelial dysfunction, and AMPK impairment act as reinforcing tributaries, ranked by evidence strength in Table 1.

## Clinical and translational implications

From a clinical perspective, the converging interaction between metabolic stress, chronic infection, and vascular dysfunction supports the concept of MASLD as a systemic cardiometabolic disorder rather than a liver-limited condition. Accumulating evidence indicates that MASLD is tightly linked to arterial hypertension and CVD, contributing not only to increased cardiometabolic risk but also to accelerated hepatic fibrosis progression and adverse long-term outcomes.^6^^,^^10^^,^^11^ This reconceptualization has direct implications for patient stratification and longitudinal monitoring, particularly in individuals with coexisting hypertension, obesity, and advanced fibrosis risk.

Arterial hypertension, beyond its frequent coexistence with MASLD, appears to exert a disease-modifying effect. Large observational and longitudinal studies demonstrate that arterial hypertension independently accelerates histological fibrosis progression and increases the risk of hepatic decompensation in MASLD.^11^^,^^52^ These data underscore the importance of systematic blood pressure assessment and aggressive antihypertensive management as potentially modifiable targets to attenuate liver-related outcomes in high-risk MASLD populations.

Beyond classical metabolic and hemodynamic stressors, chronic *Hp*-I has emerged as a clinically relevant modifier of cardiometabolic risk. Persistent *Hp-*related immune activation is associated with systemic inflammation, oxidative stress, IR, and endothelial dysfunction—mechanisms that substantially overlap with MASLD pathogenesis and hypertension onset.^7^^,^^12^ Epidemiological and mechanistic data suggest that *Hp*-I may amplify metabolic and vascular injury, particularly in patients with MASLD and established MetS, thereby contributing to disease progression and adverse outcomes.

At a translational level, AMPK signaling represents a central mechanistic node linking metabolic stress, inflammation, and vascular homeostasis. Impaired AMPK activity has been implicated in IR, oxidative stress, and hypertension,^26^ while AMPK activation confers vascular and metabolic protection.^50^ Pharmacological AMPK activation—exemplified by imeglimin targeting presenilin enhancer-2 (PEN2)—has demonstrated the capacity to alleviate hepatic lipotoxicity while exerting favorable metabolic effects.^23^ Such pleiotropic actions are particularly attractive in MASLD, a disease defined by the convergence of hepatic, metabolic, and cardiovascular dysfunction.

Collectively, these observations support an integrated, systems-based approach to MASLD management that extends beyond liver-centric strategies. Incorporating metabolic optimization, cardiovascular risk control, and—in selected patients—targeted treatment of chronic infections may offer a rational framework for modifying disease trajectory. Future prospective and interventional studies are warranted to determine whether combined metabolic, antihypertensive, and anti-infective strategies can meaningfully alter the natural history of MASLD and reduce its hepatic and extrahepatic complications.

Translating this mechanistic framework into clinical practice, several concrete diagnostic and therapeutic strategies merit consideration. Firstly, systematic screening for active *Hp*-I should be considered in patients with MASLD and coexisting MetS or its components such as arterial hypertension, obesity, diabetes, given the potential for *Hp* eradication to attenuate the inflammatory and metabolic drivers of disease progression.^32^ Secondly, noninvasive biomarkers emerging from this framework—including NAR as a composite inflammatory-metabolic index, circulating CK-18 fragments (M30/M65) for distinguishing MASH from simple steatosis, and asymmetric dimethylarginine (ADMA) as a marker of endothelial dysfunction—may offer clinically applicable tools for risk stratification and longitudinal monitoring in patients with MASLD, without requiring liver biopsy.^42^^,^^69^^,^^75^ Third, genetic profiling for the AGTR1 A1166C (rs5186) gain-of-function variant may identify a MASLD subpopulation that could derive particular benefit from personalized angiotensin receptor blocker therapy, warranting prospective evaluation.^85^ Fourth, pharmacological AMPK activation—exemplified by imeglimin targeting PEN2—represents a pleiotropic therapeutic avenue capable of simultaneously addressing hepatic lipotoxicity, insulin resistance, oxidative stress, and vascular injury, making it an attractive candidate for combination strategies in MASLD with arterial hypertension.^23^ Finally, systematic blood pressure assessment and aggressive antihypertensive management should be considered as a modifiable therapeutic target in MASLD, given the knowledge of the role of hypertension as an independent accelerator of histological fibrosis progression and hepatic decompensation.^51^

## Conclusions

MASLD is increasingly recognized as a systemic cardiometabolic disease arising from the convergence of metabolic stress, chronic low-grade inflammation, and vascular dysfunction, rather than a condition restricted solely to hepatic fat accumulation. Growing clinical and mechanistic evidence supports a bidirectional interplay between MASLD and hypertension, with the latter acting not only as a comorbidity but also as a modifier of disease progression and long-term outcomes.^51^^,^^87^

At a mechanistic level, AMPK signaling represents a central integrative axis linking metabolic stress, inflammation, and blood pressure regulation. Impaired AMPK activity contributes to IR, oxidative stress, and hypertension, while pharmacological AMPK activation—exemplified by imeglimin targeting presenilin enhancer-2—offers a promising translational avenue to simultaneously address hepatic and vascular injury.^23^

Taken together, these data argue for a paradigm shift in MASLD management toward an integrated, systems-based approach that incorporates metabolic control, cardiovascular risk modification, and—in selected patients—targeted evaluation and treatment of chronic infections. Future prospective and interventional studies are required to clarify causality and to determine whether combined metabolic, antihypertensive, and anti-infective strategies can meaningfully alter the natural history of MASLD and reduce its hepatic and extrahepatic complications.

## Data Availability

There are no new data associated with this article.

## References

[1] Furman D , Campisi J, Verdin E, et al Chronic inflammation in the etiology of disease across the life span. Nat Med. 2019;25:1822-1832. 31806905 10.1038/s41591-019-0675-0 PMC7147972

[2] Libby P. Inflammation in atherosclerosis. Nature. 2002;420:868-874. 12490960 10.1038/nature01323

[3] Lai M , Dillon ST, Gu X, et al Serum protein risk stratification score for diagnostic evaluation of metabolic dysfunction-associated steatohepatitis. Hepatol Commun. 2024;8:e0586. 10.1097/HC9.0000000000000586 PMC11608748 39621304

[4] Tincopa MA , Anstee QM, Loomba R. New and emerging treatments for metabolic dysfunction-associated steatohepatitis. Cell Metab. 2024;36:912-926. 38608696 10.1016/j.cmet.2024.03.011

[5] European Association for the Study of the Liver (EASL); European Association for the Study of Diabetes (EASD); European Association for the Study of Obesity (EASO). EASL-EASD-EASO clinical practice guidelines on the management of metabolic dysfunction-associated steatotic liver disease (MASLD). J Hepatol. 2024;81:492-542. 38851997 10.1016/j.jhep.2024.04.031

[6] Kountouras J , Papaefthymiou A, Polyzos SA, et al Impact of active *Helicobacter pylori* infection-related metabolic syndrome on systemic arterial hypertension. Arq Bras Cardiol. 2022;119:502-504. 36074383 10.36660/abc.20210931 PMC9438526

[7] Kountouras J , Papaefthymiou A, Polyzos SA, Kyrailidi F, Doulberis M. Potential impact of *Helicobacter pylori*-related metabolic syndrome on arterial hypertension outcomes. Am J Hypertens. 2023;36:192-194. 36272097 10.1093/ajh/hpac120

[8] Manolis AA , Manolis TA, Vouliotis A, Manolis AS. Metabolic dysfunction-associated steatotic liver disease and the cardiovascular system. Trends Cardiovasc Med. 2025;35:258-265. 39848507 10.1016/j.tcm.2025.01.001

[9] Polyzos SA , Orfanidou M, Kountouras J, Goulas A. Metabolic dysfunction-associated steatotic liver disease and peripheral arterial disease: associations and treatment considerations. Curr Vasc Pharmacol. 2026. 10.2174/0115701611414052251127074106 41582340

[10] Ng CH , Wong ZY, Chew NWS, et al Hypertension is prevalent in non-alcoholic fatty liver disease and increases all-cause and cardiovascular mortality. Front Cardiovasc Med. 2022;9:942753. 36003916 10.3389/fcvm.2022.942753 PMC9393330

[11] Zhao YC , Zhao GJ, Chen Z, She ZG, Cai J, Li H. Nonalcoholic fatty liver disease: an emerging driver of hypertension. Hypertension. 2020;75:275-284. 31865799 10.1161/HYPERTENSIONAHA.119.13419

[12] Kountouras J , Boziki M, Polyzos SA, et al Impact of reactive oxygen species generation on *Helicobacter pylori*-related extragastric diseases: a hypothesis. Free Radic Res. 2017;51:73-79. 28095729 10.1080/10715762.2016.1271122

[13] Thomas JA , Kendall BJ, El-Serag HB, Thrift AP, Macdonald GA. Hepatocellular and extrahepatic cancer risk in people with non-alcoholic fatty liver disease. Lancet Gastroenterol Hepatol. 2024;9:159-169. 38215780 10.1016/S2468-1253(23)00275-3

[14] Smith BK , Marcinko K, Desjardins EM, Lally JS, Ford RJ, Steinberg GR. Treatment of nonalcoholic fatty liver disease: role of AMPK. Am J Physiol Endocrinol Metab. 2016;311:E730-E740. 27577854 10.1152/ajpendo.00225.2016

[15] Zhao P , Saltiel AR. From overnutrition to liver injury: AMP-activated protein kinase in nonalcoholic fatty liver diseases. J Biol Chem. 2020;295:12279-12289. 32651233 10.1074/jbc.REV120.011356 PMC7443502

[16] Li N , Hao L, Li S, et al The NRF-2/HO-1 signaling pathway: a promising therapeutic target for metabolic dysfunction-associated steatotic liver disease. J Inflamm Res. 2024;17:8061-8083. 39512865 10.2147/JIR.S490418 PMC11542495

[17] An H , Jang Y, Choi J, Hur J, Kim S, Kwon Y. New insights into AMPK, as a potential therapeutic target in metabolic dysfunction-associated steatotic liver disease and hepatic fibrosis. Biomol Ther (Seoul). 2025;33:18-38. 39702310 10.4062/biomolther.2024.188 PMC11704404

[18] Akl MG , Li L, Widenmaier SB. Protective effects of hepatocyte stress defenders, Nrf1 and Nrf2, against MASLD progression. Int J Mol Sci. 2024;25(15):8046. 10.3390/ijms25158046 PMC11312428 39125617

[19] Bellezza I , Mierla AL, Minelli A. Nrf2 and NF-kappaB and their concerted modulation in cancer pathogenesis and progression. Cancers (Basel). 2010;2:483-497. 24281078 10.3390/cancers2020483 PMC3835087

[20] Bacon S , Seeneevassen L, Fratacci A, et al Nrf2 downregulation contributes to epithelial-to-mesenchymal transition in *Helicobacter pylori*-infected cells. Cancers (Basel). 2022;14(17):4316. 10.3390/cancers14174316 PMC9455077 36077851

[21] Bellanti F , di Bello G, Iannelli G, et al Inhibition of nuclear factor (erythroid-derived 2)-like 2 promotes hepatic progenitor cell activation and differentiation. NPJ Regen Med. 2021;6:28. 34039998 10.1038/s41536-021-00137-z PMC8155039

[22] Büyük M , Berker N, Bağbudar S, Çavuş B, Güllüoğlu M. Hepatic progenitor cell activation and ductular reaction in metabolic dysfunction-associated steatotic liver disease (MASLD): indicators for disease activity and the degree of fibrosis: the pilot study. Medicine (Baltimore). 2025;104:e42108. 40228280 10.1097/MD.0000000000042108 PMC11999437

[23] Li J , Zhang R, Zeng X, et al Imeglimin ameliorates MASLD by targeting PEN2 to activate AMPK pathway. Metabolism. 2026;175:156458. 41308829 10.1016/j.metabol.2025.156458

[24] Cheng SC , Quintin J, Cramer RA, et al mTOR- and HIF-1alpha-mediated aerobic glycolysis as metabolic basis for trained immunity. Science. 2014;345:1250684. 25258083 10.1126/science.1250684 PMC4226238

[25] Khoury MK , Yang H, Liu B. Macrophage biology in cardiovascular diseases. Arterioscler Thromb Vasc Biol. 2021;41:e77-e81. 10.1161/ATVBAHA.120.313584 PMC8046835 33054391

[26] Liang B , Wang S, Wang Q, et al Aberrant endoplasmic reticulum stress in vascular smooth muscle increases vascular contractility and blood pressure in mice deficient of AMP-activated protein kinase-alpha2 in vivo. Arterioscler Thromb Vasc Biol. 2013;33:595-604. 23288166 10.1161/ATVBAHA.112.300606 PMC3754846

[27] Persico M , Masarone M, Damato A, et al Non alcoholic fatty liver disease and eNOS dysfunction in humans. BMC Gastroenterol. 2017;17:35. 28264657 10.1186/s12876-017-0592-y PMC5340006

[28] Kountouras J. A glimpse into *Helicobacter pylori* involvement in hepatic encephalopathy. Arab J Gastroenterol. 2025;26:141-142. 39870566 10.1016/j.ajg.2025.01.005

[29] Doulberis M , Papaefthymiou A, Srivastava DS, et al Update on the association between non-alcoholic fatty liver disease and *Helicobacter pylori* infection. Int J Clin Pract. 2021;75:e13737. 32991019 10.1111/ijcp.13737

[30] Goo MJ , Ki MR, Lee HR, et al *Helicobacter pylori* promotes hepatic fibrosis in the animal model. Lab Invest. 2009;89:1291-1303. 19736546 10.1038/labinvest.2009.90

[31] Doulberis M , Srivastava S, Polyzos SA, et al Active *Helicobacter pylori* infection is independently associated with nonalcoholic steatohepatitis in morbidly obese patients. J Clin Med. 2020;9(4):933. 10.3390/jcm9040933 PMC7230908 32235601

[32] Jaber F , Alsakarneh S, Beran A, et al Impact of *Helicobacter pylori* eradication on clinical and laboratory parameters in non-alcoholic fatty liver disease patients: a systematic review and meta-analysis of randomized controlled trials. Curr Med Sci. 2025;45:1-10. 39998769 10.1007/s11596-025-00001-x

[33] Xu G , Ma S, Dong L, Mendez-Sanchez N, Li H, Qi X. Relationship of *Helicobacter pylori* infection with nonalcoholic fatty liver disease: a meta-analysis. Can J Gastroenterol Hepatol. 2023;2023:5521239. 36742347 10.1155/2023/5521239 PMC9891807

[34] Dandona P , Aljada A, Bandyopadhyay A. Inflammation: the link between insulin resistance, obesity and diabetes. Trends Immunol. 2004;25:4-7. 14698276 10.1016/j.it.2003.10.013

[35] Lorbeer R , Bayerl C, Auweter S, et al Association between MRI-derived hepatic fat fraction and blood pressure in participants without history of cardiovascular disease. J Hypertens. 2017;35:737-744. 28253218 10.1097/HJH.0000000000001245

[36] Qian LY , Tu JF, Ding YH, et al Association of blood pressure level with nonalcoholic fatty liver disease in nonhypertensive population: normal is not the new normal. Medicine (Baltimore). 2016;95:e4293. 27442673 10.1097/MD.0000000000004293 PMC5265790

[37] Li J , Verhaar AP, Pan Q, de Knegt RJ, Peppelenbosch MP. Serum levels of caspase-cleaved cytokeratin 18 (CK18-Asp396) predict severity of liver disease in chronic hepatitis B. Clin Exp Gastroenterol. 2017;10:203-209. 28860836 10.2147/CEG.S135526 PMC5560566

[38] Bar H , Bea F, Blessing E, et al Phosphorylation of cytokeratin 8 and 18 in human vascular smooth muscle cells of atherosclerotic lesions and umbilical cord vessels. Basic Res Cardiol. 2001;96:50-58. 11215532 10.1007/s003950170077

[39] Ismaiel A , Almonajjed MB, Catana CS, Popa SL, Dumitrascu DL. Metabolic dysfunction-associated steatohepatitis in focus: pathogenesis, non-invasive diagnostics, and future approaches. Arch Med Res. 2025;56:103350. 41317417 10.1016/j.arcmed.2025.103350

[40] de Alteriis G , Pugliese G, Di Sarno A, et al Visceral obesity and cytokeratin-18 antigens as early biomarkers of liver damage. Int J Mol Sci. 2023;24(13):10885. 10.3390/ijms241310885 PMC10341576 37446065

[41] Xu H , Ma X, Chen F, Zhou B. From the liver to heart in nonalcoholic fatty liver disease: single-cell and serum evidence for cytokeratin 18 in predicting cardiovascular risk. Cardiovasc Ther. 2026;2026:3055531. 41551909 10.1155/cdr/3055531 PMC12808931

[42] Zhang H , Rios RS, Boursier J, et al Hepatocyte apoptosis fragment product cytokeratin-18 M30 level and non-alcoholic steatohepatitis risk diagnosis: an international registry study. Chin Med J (Engl). 2023;136:341-350. 36848175 10.1097/CM9.0000000000002603 PMC10106257

[43] Todorovic V , Sokic-Milutinovic A, Drndarevic N, et al Expression of cytokeratins in *Helicobacter pylori*-associated chronic gastritis of adult patients infected with cagA+ strains: an immunohistochemical study. World J Gastroenterol. 2006;12:1865-1873. 16609992 10.3748/wjg.v12.i12.1865 PMC4087511

[44] Alvarez CS , Florio AA, Butt J, et al Associations between *Helicobacter pylori* with nonalcoholic fatty liver disease and other metabolic conditions in Guatemala. Helicobacter. 2020;25:e12756. 33006810 10.1111/hel.12756 PMC7688101

[45] Chen Y , You N, Shen C, Wu J, Zhang J. *Helicobacter pylori* infection increases the risk of nonalcoholic fatty liver disease in diabetic population. Front Nutr. 2023;10:1076579. 36819677 10.3389/fnut.2023.1076579 PMC9929141

[46] Yu YY , Tong YL, Wu LY, Yu XY. *Helicobacter pylori* infection eradication for nonalcoholic fatty liver disease: a randomized controlled trial. Sci Rep. 2022;12:19530. 36376474 10.1038/s41598-022-23746-0 PMC9663549

[47] Kim JY , Kwan BS, Cho JH, et al Persistently active *Helicobacter pylori* infection is associated with the development of metabolic dysfunction-associated steatotic liver disease. J Clin Med. 2025;14(4):1073. 10.3390/jcm14041073 PMC11856028 40004603

[48] Kountouras J , Doulberis M, Polyzos SA, et al Impact of *Helicobacter pylori* and/or *Helicobacter pylori*-related metabolic syndrome on incidence of all-cause and Alzheimer’s dementia. Alzheimers Dement. 2019;15:723-725. 30872115 10.1016/j.jalz.2019.01.008

[49] Kountouras J , Papaefthymiou A, Polyzos SA, et al Impact of *Helicobacter pylori*-related metabolic syndrome parameters on arterial hypertension. Microorganisms. 2021;9(11):2351. 10.3390/microorganisms9112351 PMC8618184 34835476

[50] Tain YL , Hsu CN. The impact of nutrient intake and metabolic wastes during pregnancy on offspring hypertension: challenges and future opportunities. Metabolites. 2023;13(3):418. 10.3390/metabo13030418 PMC10052993 36984857

[51] Zhou XD , Lian LY, Chen QF, et al; VCTE-Prognosis Study Group; Paired Liver Biopsy Group. Effect of hypertension on long-term adverse clinical outcomes and liver fibrosis progression in MASLD. J Hepatol. 2026;84:254-265. 40854336 10.1016/j.jhep.2025.08.017

[52] Barrett R , Archer A, Cathcart J, Abeysekera K, Dillon JF, Brennan PN. Progression to decompensation of severe fibrosis compared to cirrhosis in MASLD: a systematic review and meta-analysis. Liver Int. 2026;46:e70511. 41546486 10.1111/liv.70511 PMC12811796

[53] Fabbrini E , Sullivan S, Klein S. Obesity and nonalcoholic fatty liver disease: biochemical, metabolic, and clinical implications. Hepatology. 2010;51:679-689. 20041406 10.1002/hep.23280 PMC3575093

[54] Nosalski R , Guzik TJ. Perivascular adipose tissue inflammation in vascular disease. Br J Pharmacol. 2017;174:3496-3513. 28063251 10.1111/bph.13705 PMC5610164

[55] Yu Y , Cai J, She Z, Li H. Insights into the epidemiology, pathogenesis, and therapeutics of nonalcoholic fatty liver diseases. Adv Sci (Weinh). 2019;6:1801585. 30828530 10.1002/advs.201801585 PMC6382298

[56] Dogan Z , Sarikaya M, Ergul B, Filik L. The effect of *Helicobacter pylori* eradication on insulin resistance and HbA1c level in people with normal glucose levels: a prospective study. Biomed Pap Med Fac Univ Palacky Olomouc Czech Repub. 2015;159:242-245. 24993741 10.5507/bp.2014.036

[57] Ferrara D , Montecucco F, Dallegri F, Carbone F. Impact of different ectopic fat depots on cardiovascular and metabolic diseases. J Cell Physiol. 2019;234:21630-21641. 31106419 10.1002/jcp.28821

[58] Kountouras J , Boziki M, Polyzos SA, et al The emerging role of *Helicobacter pylori*-induced metabolic gastrointestinal dysmotility and neurodegeneration. Curr Mol Med. 2017;17:389-404. 29256351 10.2174/1566524018666171219094837

[59] Leung C , Rivera L, Furness JB, Angus PW. The role of the gut microbiota in NAFLD. Nat Rev Gastroenterol Hepatol. 2016;13:412-425. 27273168 10.1038/nrgastro.2016.85

[60] Fukuda Y , Bamba H, Okui M, et al *Helicobacter pylori* infection increases mucosal permeability of the stomach and intestine. Digestion. 2001;63(Suppl 1):93-96. 11173917 10.1159/000051918

[61] Tripathi A , Debelius J, Brenner DA, et al The gut-liver axis and the intersection with the microbiome. Nat Rev Gastroenterol Hepatol. 2018;15:397-411. 29748586 10.1038/s41575-018-0011-z PMC6319369

[62] Kountouras J , Zavos C, Polyzos SA, Deretzi G. Potential impact of *Helicobacter pylori*-related human beta-defensin-1 on hepatic encephalopathy and neurodegeneration. Ann Gastroenterol. 2016;29:99. PMC4700858 26752958

[63] Sharifnia T , Antoun J, Verriere TG, et al Hepatic TLR4 signaling in obese NAFLD. Am J Physiol Gastrointest Liver Physiol. 2015;309:G270-G278. 26113297 10.1152/ajpgi.00304.2014 PMC4537925

[64] Li Z , Zhang F, Sun M, et al The modulatory effects of gut microbes and metabolites on blood-brain barrier integrity and brain function in sepsis-associated encephalopathy. PeerJ. 2023;11:e15122. 37009158 10.7717/peerj.15122 PMC10064995

[65] Tsukamoto H , Mizoshita T, Sasaki M, et al Long-term high-dose proton pump inhibitor administration to *Helicobacter pylori*-infected Mongolian gerbils enhances neuroendocrine tumor development in the glandular stomach. Asian Pac J Cancer Prev. 2011;12:1049-1054. 21790250

[66] Shahzad A , Ni Y, Yang Y, et al Neutrophil extracellular traps (NETs) in health and disease. Mol Biomed. 2025;6:130. 41335221 10.1186/s43556-025-00337-9 PMC12675906

[67] Shrestha S , Jeon JH, Hong CW. Neutrophils in MASLD and MASH. BMB Rep. 2025;58:116-123. 39757200 10.5483/BMBRep.2024-0058 PMC11955729

[68] Bao B , Xu S, Sun P, Zheng L. Neutrophil to albumin ratio: a biomarker in non-alcoholic fatty liver disease and with liver fibrosis. Front Nutr. 2024;11:1368459. 38650638 10.3389/fnut.2024.1368459 PMC11033504

[69] Liu CF , Chien LW. Predictive role of neutrophil-percentage-to-albumin ratio (NPAR) in nonalcoholic fatty liver disease and advanced liver fibrosis in nondiabetic US adults: evidence from NHANES 2017-2018. Nutrients. 2023;15(8):1892. 10.3390/nu15081892 PMC10141547 37111111

[70] Fan M , Song E, Zhang Y, et al Metabolic dysfunction-associated steatohepatitis detected by neutrophilic crown-like structures in morbidly obese patients: a multicenter and clinicopathological study. Research (Wash D C). 2024;7:0382. 38812532 10.34133/research.0382 PMC11134285

[71] Chen X , Peng R, Peng D, Liu D, Li R. *Helicobacter pylori* infection exacerbates metabolic dysfunction-associated steatotic liver disease through lipid metabolic pathways: a transcriptomic study. J Transl Med. 2024;22:701. 39075482 10.1186/s12967-024-05506-y PMC11288106

[72] Liu A , Deng X, Hou S, Xi Y, Xu K. Activated immune and complement C3 are potential contributors in MASH via stimulating neutrophil extracellular traps. Cells. 2025;14(10):740. 10.3390/cells14100740 PMC12110206 40422243

[73] He L , Yang H, Zeng Z, Wang Q, Guan H, Zhao P. *Helicobacter pylori* infection and its impact on metabolic dysfunction-associated steatotic liver disease: a mediation analysis of neutrophil-albumin ratio. Front Nutr. 2025;12:1701544. 41459074 10.3389/fnut.2025.1701544 PMC12739950

[74] Yola IM , Moser C, Duncan MS, et al Associations of circulating dimethylarginines with the metabolic syndrome in the Framingham Offspring study. PLoS One. 2021;16:e0254577. 34492019 10.1371/journal.pone.0254577 PMC8423279

[75] Dogru T , Genc H, Tapan S, et al Elevated asymmetric dimethylarginine in plasma: an early marker for endothelial dysfunction in non-alcoholic fatty liver disease? Diabetes Res Clin Pract. 2012;96:47-52. 22189171 10.1016/j.diabres.2011.11.022

[76] Lewinska A , Wnuk M. *Helicobacter pylori*-induced premature senescence of extragastric cells may contribute to chronic skin diseases. Biogerontology. 2017;18:293-299. 28074309 10.1007/s10522-017-9676-x PMC5350214

[77] Cai Q , Shi P, Yuan Y, et al Inflammation-associated senescence promotes *Helicobacter pylori*-induced atrophic gastritis. Cell Mol Gastroenterol Hepatol. 2021;11:857-880. 33161156 10.1016/j.jcmgh.2020.10.015 PMC7859172

[78] Mastoridou EM , Goussia AC, Kataki A, et al The interplay between cellular senescence and lipid metabolism in the progression of metabolic dysfunction-associated steatotic liver disease (MASLD). Int J Mol Sci. 2026;27(2):1066. 10.3390/ijms27021066 PMC12842010 41596708

[79] Wijayasiri P , Astbury S, Kaye P, et al Role of hepatocyte senescence in the activation of hepatic stellate cells and liver fibrosis progression. Cells. 2022;11(14):2221. 10.3390/cells11142221 PMC9322633 35883664

[80] Pasarin M , La Mura V, Gracia-Sancho J, et al Sinusoidal endothelial dysfunction precedes inflammation and fibrosis in a model of NAFLD. PLoS One. 2012;7:e32785. 22509248 10.1371/journal.pone.0032785 PMC3317918

[81] Oshima T , Ozono R, Yano Y, et al Association of *Helicobacter pylori* infection with systemic inflammation and endothelial dysfunction in healthy male subjects. J Am Coll Cardiol. 2005;45:1219-1222. 15837252 10.1016/j.jacc.2005.01.019

[82] Carr RM , Oranu A, Khungar V. Nonalcoholic fatty liver disease: pathophysiology and management. Gastroenterol Clin North Am. 2016;45:639-652. 27837778 10.1016/j.gtc.2016.07.003 PMC5127277

[83] Banini BA , Sanyal AJ. Nonalcoholic fatty liver disease: epidemiology, pathogenesis, natural history, diagnosis, and current treatment options. Clin Med Insights Ther. 2016;8:75-84. 28670148 10.4137/cmt.s18885 PMC5491796

[84] Maiorana F , Neschuk M, Caronia MV, et al The interplay between *Helicobacter pylori* infection and rs738409 PNPLA3 in metabolic dysfunction-associated steatotic liver disease. PLoS One. 2024;19:e0310361. 39312529 10.1371/journal.pone.0310361 PMC11419387

[85] Li L , Liu H, Hu X, et al Identification of key genes in non‑alcoholic fatty liver disease progression based on bioinformatics analysis. Mol Med Rep. 2018;17:7708-7720. 29620197 10.3892/mmr.2018.8852 PMC5983972

[86] Li Y , Xu H, Wu W, et al Clinical application of angiotensin receptor blockers in patients with non-alcoholic fatty liver disease: a systematic review and meta-analysis. Oncotarget. 2018;9:24155-24167. 29844879 10.18632/oncotarget.23816 PMC5963622

[87] Gao Z , Deng H, Qin B, Bai L, Li J, Zhang J. Impact of hypertension on liver fibrosis in patients with metabolic dysfunction-associated fatty liver disease. Front Med (Lausanne). 2025;12:1539283. 39911867 10.3389/fmed.2025.1539283 PMC11794791

